# L’apport de l’échographie dans l’exploration des métrorragies post-ménopausiques

**DOI:** 10.11604/pamj.2016.24.175.7361

**Published:** 2016-06-30

**Authors:** Fethia Boudaya, Amel Achour Jenayah, Sarah Saoudi, Anissa Gharsa, Eya Gharbi, Ezzeddine Sfar, Dalenda Chelli

**Affiliations:** 1 Service de Gynécologie Obstétrique «A», Centre de Maternité et de Néonatologie de Tunis, Tunisie

**Keywords:** Métrorragies, ménopause, hystéroscopie, histologie, lésion organique, Metrorrhagias, hysteroscopy, histology, organic lesion

## Abstract

Les métrorragies post-ménopausiques représentant un motif fréquent de consultation en gynécologie. Notre étude avait pour but d’évaluer l’apport de l’échographie pelvienne dans l’exploration des lésions endo-cavitaires devant ce symptôme et de comparer les résultats retrouvés à ceux de l’hystéroscopie et de l’histologie. Il s’agissait d’une étude rétrospective analytique à propos de 33 observations de patientes prises en charge pour des métrorragies post-ménopausiques au service de gynécologie obstétrique «A» du centre de maternité et de néonatologie de Tunis durant l’année 2012. Toutes les patientes avaient bénéficié d’une échographie pelvienne et d’une hystéroscopie diagnostique. Nous avons analysé et confronté les données de l’échographie pelvienne, de l’hystéroscopie et de l’examen histologique. L’âge moyen de nos patientes était de 57,78 ans et l'âge moyen de la ménopause était de 48,36 ans. La confrontation des données échographiques et histologiques, a montré que l’échographie a présenté une sensibilité de 80,6%, une spécificité de 79,38%, une valeur prédictive positive (VPP) de l’ordre de 67,03% et une valeur prédictive négative (VPN) de 91,54%. Quant à l’hystéroscopie, ces valeurs étaient respectivement de 84,44%, 82,72%, 69,93% et 92,65%. Le degré de performance de chaque moyen d’exploration a été variable selon la lésion en cause des métrorragies et d’une façon générale l’hystéroscopie a été plus fiable dans l’exploration des métrorragies que l’échographie: indice de Youden 0,67 contre 0,59. Nos résultats rejoignent les données de la littérature qui attribue à l’hystéroscopie une plus grande fiabilité par rapport à l’échographie pelvienne dans le diagnostic des lésions endo-cavitaires à l’origine des métrorragies post-ménopausiques.

## Abstract (en)

Post-menopausal metrorrhagias represent a frequent reason for consultation in gynecology. Our study aims to evaluate the contribution of pelvic ultrasonography in the exploration of endocavitary lesions in people experiencing this symptom and to compare the results found with those of hysteroscopy and histology. This was an analytic retrospective study of 33 cases referred for evaluation of postmenopausal metrorrhagias at the department of gynecology and obstetrics «A» in the Center of Maternity and Neonatology of Tunis in 2012. All patients underwent pelvic ultrasonography and diagnostic hysteroscopy. We analyzed and compare the data obtained with pelvic ultrasonography, hysteroscopy and histological examination. The average age of our patients was 57.78 years and the average age of menopause was 48.36 years. The confrontation between ultrasonographic and histological data showed that ultrasonography has a sensitivity of 80.6%, a specificity of 79.38%, positive predictive value (PPV) of 67.03% and negative predictive value (NPV) of 91.54%. With respect to hysteroscopy these values were 84,44%, 82,72%, 69,93% and 92,65% respectively. Performance level for each exploratory diagnostic tool varied according to the lesion which caused metrorrhagias and generally hysteroscopy was more reliable in the exploration of metrorrhagias than ultrasonography: Youden index 0.67 against 0.59. Our results confirmed data published in the literature that assigns to hysteroscopy a greater reliability compared to pelvic ultrasonography in the diagnosis of endocavitary lesions causing postmenopausal metrorrhagias.

## Introduction

Les Métrorragies post-Ménopausiques représentent un motif fréquent de consultation en gynécologie. En effet, près de 70% des consultations de la femme en péri ou post-ménopause se rapportent à des saignements [1] et 5% de toutes les investigations gynécologiques concernent les métrorragies post ménopausiques [2]. Cette problématique tend à s’accentuer en raison du vieillissement de la population, de l’usage plus fréquent de la substitution hormonale et de la prescription du tamoxifène dans le cadre des tumeurs mammaires malignes [3, 4]. Elles constituent le principal signal d’alarme pour un carcinome de l’endomètre [5]. L’échographie pelvienne réalisée notamment par voie endo-vaginale représente un moyen d’investigation anodin et facilement disponible pour explorer l’utérus et les annexes. En cas d’anomalies échographiques, une hystéroscopie diagnostique est envisagée. Elle permettra une biopsie dirigée de l’endomètre et/ou de toute lésion endocavitaire. Le but de notre travail était d’évaluer l’apport de l’échographie pelvienne dans l’exploration des métrorragies post-ménopausiques.

## Méthodes

Il s’agissait d’une étude rétrospective ayant colligé des patientes prises en charge pour des métrorragies post ménopausiques au service “A” du centre de maternité et de néonatalogie de Tunis, durant toute l’année 2012. Nous avons inclus dans notre étude les patientes qui ont consulté pour des métrorragies post ménopausiques et qui ont bénéficié d’une échographie pelvienne couplée à une hystéroscopie diagnostique avec biopsie.

Les données ont été dans un premier temps saisies dans des grilles. L’analyse a été effectuée par le logiciel Excel. Pour évaluer la performance des techniques utilisées à visée diagnostique, nous avons utilisé les paramètres de validité internes: la sensibilité, la spécificité, la valeur prédictive positive (VPP), la valeur prédictive négative (VPN). Ces paramètres étaient exprimés en pourcentage. Pour évaluer l’efficacité des techniques utilisées nous avons calculé l’indice de Youden: J = sensibilité + spécificité – 1; indice négatif = test inefficace; indice se rapprochant de 1 = test efficace.

## Résultats

### Profil épidémiologique des patientes

Durant l’année 2012, nous avons colligé 33 patientes consultant pour des métrorragies post ménopausiques. L’âge moyen de nos patientes était de 57,78 ans avec des extrêmes allant de 46 à 76 ans. Dix patientes (30,3%) avaient un âge supérieur à 60 ans. L´âge moyen de la ménarche était de 11,9 ans avec des extrêmes allant de 10 à 16 ans. La gestité moyenne était de 5,63 avec des extrêmes allant de 0 à 11. Deux patientes étaient nulligestes. L´âge moyen de la ménopause était de 48,36 ans avec des extrêmes allant de 40 à 56 ans. Une ménopause tardive (au-delà de 55 ans) était retrouvée chez 4 patientes.

### L’examen gynécologique

A l’examen clinique un saignement d’origine endo-utérine était noté chez 9 patientes. Le col était macroscopiquement sain chez 28 patientes. Une exocervicite a été retrouvée dans 5 cas. Le touché vaginal a objectivé un utérus augmenté de taille dans 3 cas (9%) et un comblement partiel des culs de sac latéraux dans 1 cas.

### Examens complémentaires

L’échographie pelvienne est un moyen facile et rapide pour explorer l’utérus et les annexes.

#### Echographie pelvienne

L’épaisseur moyenne de l’endomètre était de 8,36 mm avec des extrêmes allant de 3 à 19 mm. Selon les données échographiques, les métrorragies post ménopausiques étaient associées à un endomètre hypertrophié (épaisseur > 5 mm) dans 63,63% des cas. L’échographie a objectivé un utérus augmenté de taille chez 5 patientes (15,15%) en rapport avec des fibromes interstitiels dont le plus grand axe était de 50 mm (ces patientes étaient connues porteuses d’utérus polymyomateux avant la ménopause). Une image intra cavitaire a été suspectée dans 9 cas, ces images évoquaient un polype dans 8 cas et une lésion suspecte chez une patiente (image hétérogène de 20×15 mm de diamètre associée à un endomètre épais à 12 mm.

#### Hystéroscopie

L’hystéroscopie a conclu à un aspect atrophique de la muqueuse utérine chez 14 patientes soit 42,42% des cas. L’atrophie était associée à un polype endométrial chez 3 patientes. Un endomètre hypertrophié a été trouvé chez 17 patientes soit 51,15% des cas. Il était associé à un polype chez 9 patientes. Une hypertrophie endométriale associée à une lésion intra cavitaire fortement suspecte à type de formation végétante saignante au contact a été notée chez deux patientes.

#### Examen histologique

L’examen anatomo-pathologique a conclu à: un polype endométrial dans 6 cas (18.18%), un endomètre atrophique dans 11 cas (33,33%). Une hyperplasie endométriale a été notée dans 15 cas (45,46%) soit l’aspect le plus fréquent dont 8 cas correspondaient à une hyperplasie polypoide. Une hyperplasie complexe avec atypie a été notée dans un seul cas. L’examen anatomo-pathologique définitif de la pièce d’hystérectomie a conclu à un adénocarcinome de l’endomètre. C’était une patiente âgée de 57 ans, hypertendue, multipare G6 P5, ménopausée à l’âge de 50 ans, chez qui l’échographie a objectivé un utérus augmenté de taille, un endomètre de 12 mm d’épaisseur avec présence d’une image endo-cavitaire suspecte.

### Corrélation échographie- hystéroscopie

Parmi les 15 cas ayant un endomètre fin à l’échographie, 14 ont été confirmés à l’hystéroscopie, soit une concordance de 90%. Concernant les 14 cas d’endomètre épais observés à l’échographie, l’hystéroscopie en a montré 6 cas d’hypertrophie endomètriale, 6 cas de polype endomètrial, 1 cas d’atrophie endométriale, et une lésion suspecte, soit une concordance de 42,8% (Tableau 1).

**Tableau 1:** Corrélation échographie-hystéroscopie

		Hystéroscopie				
		Endomètre atrophié	Endomètre épais	Endomètre atrophié + polype	Endomètre épais + polype	Endomètre suspect
**Echographie**	**N**	11	8	3	9	2
Endomètre fin	10	9	1	0	0	0
Endomètre épais	14	1	6	2	4	1
Endomètre fin+ image intra-cavitaire	2	1	0	1	0	0
Endomètre épais+ image intra-cavitaire	7	0	1	0	5	1

### Corrélation échographie- histologie

Parmi les 10 cas jugés endomètre fin à l’échographie 9 ont été confirmés à l’histologie, soit une concordance de 90% et pour les 14 cas considérés à l’échographie comme endomètre épais, 11 cas ont été confirmés à l’histologie, soit une concordance de 81.3%.

### Corrélation hystéroscopie- histologie

Les 11 cas d’atrophie de l’endomètre diagnostiqués à l’hystéroscopie ont été confirmés à l’examen histologique, soit une concordance de 100%. Parmi les 8 cas jugés comme hypertrophie endométriale à l’hystéroscopie, 6 ont été confirmés à l’histologie soit une concordance de 75%. Parmi les 12 cas de polypes vus à l’hystéroscopie, 4 ont été confirmés histologiquement soit une concordance de 33,33% (Tableau 2). La performance de l’échographie endovaginale était variable avec le type de la lésion endo-utérine sous-jacente; les résultats sont représentés dans le Tableau 3. Le Tableau 4 illustre les paramètres statistiques utilisés pour évaluer la performance de l’hystéroscopie dans la détection des pathologies endométriales rencontrées. La corrélation hystéroscopie diagnostique-histologie était variable selon l’étiologie: une concordance de 100% pour le diagnostic d’atrophie endométriale (J = 1); une concordance modérée en cas d’hyperplasie endomètriale (J=0,64); une faible concordance en cas d’image intra-cavitaire en particulier de polype de l’endomètre (J=0,37).

**Tableau 2:** Corrélation hystéroscopie-histologie

		Histologie			
		Endomètre atrophique	Endomètre hyperplasique	Polype	Adénocarcinome
**Echographie**	**N**	11	15	6	1
Endomètre fin	11	11	0	0	0
Endomètre épais	8	0	6	2	0
Endomètre atrophié+ polype	3	0	1	2	0
Endomètre épais+ polype	9	0	7	2	0
Endomètre suspect	2	0	1	0	1

**Tableau 3:** Etude de la performance de l’échographie

Pathologie	Sensibilité (%)	Spécificité (%)	VPP (%)	VPN (%)	J
Atrophie	81,81	95,45	90	91,3	0,77
Hyperplasie	93,93	61,11	66,66	91,66	0,54
polype	66,66	81,48	44,44	91,66	0,48
Moyenne	80,6	79,38	67,03	91,54	0,59

**Tableau 4:** Etude de la performance de l’hystéroscopie

Pathologie	Sensibilité (%)	Spécificité (%)	VPP (%)	VPN (%)	J
Atrophie	100	100	100	100	1
Hyperplasie	86,66	77,77	76,47	87,5	0,64
polype	66,66	70,37	33,33	90,47	0,37
Moyenne	84,44	82,71	69,93	92,65	0,67

## Discussion

Les métrorragies constituent un motif de consultation fréquent des femmes ménopausées. Elles doivent impérativement faire rechercher une pathologie organique en cause et en particulier le cancer de l’endomètre.

### Apport de l’échographie pelvienne

L’échographie pelvienne constitue l’examen de première intention permettant d’exclure une pathologie endométriale en cas de métrorragies post-ménopausiques. Dans la littérature la sensibilité, la spécificité, la VPP et la VPN de l’échographie pelvienne dans la détection des pathologies endométriales sont variables selon les auteurs [6–12]. Dans notre étude, l’échographie semble assez performante avec une sensibilité et une spécificité respectivement de 80,6% et 79,38%. Elle s’est avérée particulièrement performante lorsqu’il s’agissait d’une atrophie (Tableau 5).

**Tableau 5:** Sensibilité, Spécificité et VPP de l’échographie endovaginale pour le diagnostic des étiologies des métrorragies post ménopausiques (notre série en comparaison à différentes études sur le sujet)

Auteurs	Année	Nombre de cas	Sensibilité (%)	Spécificité (%)	VPP (%)
Notre étude	2012	33	80,6	79.38	67,03
Furquhar et al (6)	2003	19	46 - 100	12- 100	51,5
Bosch et al (7)	1995	140	98,2	44,3	58,5
Gupta et al (8)	1996	76	83	77	54
Cacciatore et al (9)	1994	45	73, 9	95,7	94,4
Emanuel et al (10)	1995	279	96	89	-
Tawbin et al (11)	1996	149	54	90	-
Smith-Bindman et al (12)	1998	5892	66 -100	38-90	-

Cependant, l’utilisation de l’échographie pelvienne présente certaines limites dans l’exploration des métrorragies post-ménopausiques. En fait dans certains cas, elle objective un épaississement diffus de l’endomètre mais elle ne permet pas la distinction entre une hyperplasie, un polype, une lésion suspecte d’un endomètre normal. On ne peut pas aussi différencier entre un fibrome inférieur à 2 cm et un polype et si la cavité utérine est déformée par des fibromes ou des polypes, on ne peut pas mesurer l’épaisseur de l’endomètre. Pour ces raisons certaines investigations complémentaires sont souvent demandées en complément d’examen échographique anormal. Ainsi l’échographie de dépistage est considérée comme un moyen de dépistage et non de diagnostic des pathologies endométriales.

#### Etude de l’épaisseur de l’endomètre

L’étude de l’endomètre constitue une étape importante dans l’exploration des métrorragies post ménopausiques. En effet, chez une femme ménopausée une mesure de l’épaisseur de l’endomètre peut faire la part entre patientes à haut ou bas risque de cancer de l’endomètre. Certains auteurs recommandent une valeur seuil de l’épaisseur de l’endomètre de 5 mm pour les explorations étiologiques des métrorragies post-ménopausiques. Ceux-ci avaient conclu qu’en cas d’épaisseur de l’endomètre inférieure à 5 mm, il n’est pas nécessaire de faire des examens complémentaires. Dans l’étude multicentrique de Granberg [13], comprenant 1168 femmes, les 114 femmes avec un cancer de l’endomètre et les 112 femmes avec une hyperplasie avaient une épaisseur endométriale supérieure à 5 mm. Il en découle que cette valeur était admise comme valeur seuil par la majorité des études pour parler d’endomètre normal ou pathologique.

### Apport de l’hystéroscopie

L´hystéroscopie est la technique d´exploration endocavitaire la plus fiable, offrant une vision directe de la cavité utérine et permettant de faire un bilan lésionnel précis et des biopsies dirigées. Selon notre travail, l’hystéroscopie est très performante dans le diagnostic des atrophies de l’endomètre, avec une sensibilité de 100%. Cette performance est modérée en cas d’hyperplasie ou de polype de l’endomètre avec une sensibilité respectivement de 86,66% et 66,66%.

## Conclusion

L’échographie pelvienne est l’examen recommandé en première intention dans l’exploration des métrorragies post-ménopausiques. En dehors d’une atrophie endométriale, il faudrait la coupler à l’hystéroscopie, afin d’augmenter la sensibilité dans la détection des lésions endo-cavitaires.

### Etat des connaissances actuelles sur le sujet

- La prise en charge d’une patiente présentant des métrorragies post ménopausiques suppose toujours d’éliminer une étiologie organique. Le contexte doit être pris en compte, notamment la chronologie du saignement par rapport à l’installation de la ménopause;
- En début de ménopause, la pathologie est dominée par la poursuite de processus présents en pré-ménopause. Plus tardivement se manifestent à la fois les pathologies endométriales sévères (cancer et hyperplasie), mais aussi l’atrophie endométriale liée à la privation hormonale;
- Il est indispensable de réaliser des explorations poussées et notamment une hystéroscopie diagnostique lorsque le terrain fait évoquer un terrain d’hyperœstrogénie, ou si l’échographie n’est pas affirmative.

### Contribution de notre étude à la connaissance

- Notre étude a étudié de façon distincte la sensibilité, la spécificité, les valeurs prédictives positives et négatives de l’échographie pelvienne et de l’hystéroscopie dans l’exploration des métrorragies post-ménopausiques;
- Le degré de performance de chaque moyen d’exploration a été variable selon la lésion en cause des métrorragies et d’une façon générale l’hystéroscopie a été plus fiable dans l’exploration des métrorragies que l’échographie;
- Pour les équipes disposant facilement soit de l’hystéroscopie diagnostique, soit de l’hystérosonographie, ces examens seront réalisés systématiquement après l’échographie vaginale.
